# Prediction of the short-term efficacy of anti-VEGF therapy for neovascular age-related macular degeneration using optical coherence tomography angiography

**DOI:** 10.1186/s40662-022-00287-1

**Published:** 2022-05-01

**Authors:** Huixun Jia, Bing Lu, Zhi Zhao, Yang Yu, Fenghua Wang, Minwen Zhou, Xiaodong Sun

**Affiliations:** 1 Department of Ophthalmology, Shanghai General Hospital (Shanghai First People’s Hospital), Shanghai Jiao Tong University School of Medicine, 100 Hai Ning Road, Shanghai, 200080 People’s Republic of China; 2 Shanghai Key Laboratory of Ocular Fundus Diseases, Shanghai, China; 3 National Clinical Research Center for Eye Diseases, Shanghai, China; 4 Shanghai Engineering Center for Visual Science and Photomedicine, Shanghai, China; 5 Shanghai Engineering Center for Precise Diagnosis and Treatment of Eye Diseases, Shanghai, China; 6 Clinical Research Institute, Shanghai Jiao Tong University School of Medicine, Shanghai, China

**Keywords:** Age-related macular degeneration, Choroidal neovascularization, Optical coherence tomography angiography, Anti-VEGF

## Abstract

**Background:**

To evaluate whether the specific choroidal neovascularization (CNV) characteristics measured using optical coherence tomography angiography (OCTA) can predict the 6-month prognosis of neovascular age-related macular degeneration (nAMD) after anti-vascular endothelial growth factor (anti-VEGF) therapy.

**Methods:**

Patients with type 1, type 2, or mixed-type neovascularization (NV) were prospectively included. Participants underwent an initial loading phase of three consecutive monthly intravitreal injections of Conbercept (0.5 mg) and were switched to a pro re nata (PRN) treatment strategy. OCTA images were evaluated for eyes that underwent follow-up assessments for more than 6 months. CNV lesions were manually segmented, and the CNV area, vessel area, greatest vascular caliber (GVC), and greatest linear dimension (GLD) were compared between responders and non-responders. Two masked graders independently measured the above-mentioned parameters using OCTA, and consistency was assessed using the intraclass correlation coefficient (ICC) values. Multiple logistic regression analysis was performed to evaluate the effect of a 3-month change in the CNV area, GLD, and GVC on the 6-month response to anti-VEGF agents.

**Results:**

Among the 60 eyes of 60 patients with nAMD, 39 were responders and 21 were non-responders. The proportion of CNV types was significantly different between responders and non-responders (*P* = 0.009). Patients with type 2 or mixed NV seemed more likely to respond to the treatment (28.2% *vs.* 0.0%, and 30.8% *vs.* 23.8%, respectively). The change in GVC showed a significant difference between responders (− 4.98 ± 17.17 μm) and non-responders (11.01 ± 14.10 μm) after three monthly intravitreal anti-VEGF injections. Multiple logistic regression analysis showed that only the change in GVC remained significant after controlling for baseline GVC, injection number, and CNV type (adjusted OR = 1.083; *P* = 0.008).

**Conclusions:**

Type 2 and mixed-type NV were significantly associated with a better response to anti-VEGF therapy. Changes in GVC after 3 months of treatment were significantly associated with a response to anti-VEGF therapy at 6 months.

## Background

Neovascular age-related macular degeneration (nAMD) is the leading cause of irreversible vision loss in people over 50 years of age worldwide [1]. In the past decade, intravitreal anti-vascular endothelial growth factor (anti-VEGF) drugs have significantly improved the outcomes of eyes with nAMD [2–4]. However, many patients experience a poor response to anti-VEGF therapy, which presents as persistent intraretinal, subretinal, or subretinal pigment epithelial fluid, persistent or new hemorrhage, progressive lesion fibrosis, and suboptimal vision recovery [5]. Thus, there is a need to determine the factors that influence the prognosis of nAMD patients after anti-VEGF therapy in clinical practice [6–8].

At present, the potential factors associated with the prognosis of nAMD after anti-VEGF therapy are primarily based on baseline clinical characteristics, such as age [9, 10], baseline visual acuity (VA) [11, 12], and the choroidal neovascularization (CNV) lesion area [13], as well as several optical coherence tomography (OCT) characteristics such as the status of the external limiting membrane (ELM) and ellipsoid zone (EZ) [14]. Differences in these responses and clinical features may influence the choice of therapeutic regimens for ophthalmologists. Other OCT characteristics, such as the presence of pigment epithelial detachmentand drusen volume at baseline, also appear to be important predictors of vision outcomes among patients with nAMD [15].

Optical coherence tomography angiography (OCTA) allows noninvasive visualization and qualitative assessment of CNV [16–18]. Several studies have shown that the use of OCTA can facilitate evaluation of the morphological characteristics of different subtypes of CNV in eyes with nAMD [19]. In addition, certain parameters, such as greatest vascular caliber (GVC) [20, 21] and greatest linear dimension (GLD) [22], can be qualitatively measured by OCTA, and may be potential factors influencing the prognosis of nAMD after anti-VEGF therapy.

However, the ability of these CNV parameters observed on OCTA to predict the prognosis of nAMD after anti-VEGF therapy remains controversial [23–25]. Thus, to gain further insight into the potential factors that influence the prognosis of nAMD after anti-VEGF therapy, we investigated the association between the response to anti-VEGF therapy and the OCTA characteristics in patients with naïve CNV secondary to exudative AMD.

## Materials and methods

### Participants

This cohort study was approved by the ethics committee of Shanghai General Hospital (2016–021) and conducted in accordance with the tenets of the Declaration of Helsinki. Informed consent was obtained from each participant. Participants were recruited from the practices of three retina specialists (XDS., FHW, and SQY) at the Department of Ophthalmology, Shanghai General Hospital, between January 1, 2016, and December 31, 2017. Inclusion criteria were as follows: (1) patient age greater than 50 years; (2) type 1, type 2, or mixed-type neovascularization (NV) diagnosed in multimodal imaging [spectral-domain OCT (SD-OCT); Heidelberg Engineering, Heidelberg, Germany], wherein type 1 NV was defined as NV located beneath the retinal pigment epithelium (RPE) monolayer and above Bruch’s membrane; type 2 NV was defined as NV located above the RPE monolayer; and mixed-type NV was defined as a combination of type 1 and type 2 NV; (3) no prior treatment or active CNV according to SD-OCT and/or fluorescein angiography (FA) features. Only one eye of each patient was included. The exclusion criteria were as follows: (1) CNV that had received any previous treatment or was at the quiescent stage; (2) other phenotypes of exudative AMD, such as type 3 NV [retinal angiomatous proliferation (RAP)] or polypoidal choroidal vasculopathy (PCV); (3) presence of geographic atrophy; (4) presence of myopia < −6 D, evidence of pathologic myopia or any other retinal diseases, including angioid streaks, central serous chorioretinopathy, or diabetic retinopathy; (5) poor imaging data in OCTA (i.e., due to media opacity and eye movement) or a CNV lesion that exceeded the 3 × 3 mm scanning area.

### Intervention and data acquisition

Participants underwent an initial loading phase consisting of three consecutive monthly intravitreal injections of Conbercept (0.5 mg) and were then switched to a pro re nata (PRN) treatment strategy. The participants underwent a comprehensive ophthalmic examination, including best-corrected visual acuity (BCVA) measurement using the Early Treatment Diabetic Retinopathy Study (ETDRS) chart, dilated examinations with fundus photography, FA, indocyanine green angiography (ICGA; Heidelberg Retina Angiograph II; Heidelberg Engineering, Heidelberg, Germany), SD-OCT (Spectralis; Heidelberg Engineering, Heidelberg, Germany), and OCTA (Optovue RTVue XR Avanti; Optovue, Inc., Fremont, California, USA) on the same day. BCVA measurements, SD-OCT, and OCTA were performed at each visit. FA and ICGA were performed before initial anti-VEGF injections. The duration of symptoms was determined according to patients’ reports. All patients underwent follow-up evaluations for at least 6 months after the initial injection.

SD-OCT scans were performed as follows: horizontal volume scan (19 sections), macular star (6 sections), and a horizontal follow-up 6 mm scan. Central retinal thickness (CRT) was defined as the distance between the internal limiting membrane and the presumed RPE at the fovea by measuring the volume scan after exact centering of the scan and alignment of the automated lines.

The instrument used for the OCTA images was based on the Optovue RTVue XR Avanti. This system utilizes an A-scan rate of 70,000 scans per second, with a light source centered at 840 nm and a bandwidth of 45 nm, to obtain split-spectrum amplitude decorrelation angiography images. A 3 × 3 mm scan area was chosen to capture the entire CNV lesion.

### Definition of response to anti-VEGF therapies

Response to anti-VEGF therapies in nAMD was classified as response or non-response according to the guidelines proposed by Amoaku et al. [23], which were based primarily on morphological changes; functional changes were also included. Responders were defined as patients who achieved a VA improvement of ≥ 5 ETDRS letters, a CRT reduction > 25%, or alleviation of subretinal fluid, intraretinal fluid, or intraretinal cysts at 6 months. Non-responders were defined as patients who did not meet these criteria. Six months after the baseline, response to anti-VEGF therapies was independently assessed by two masked graders (BL and FY) based on VA and OCT data, including evaluations of CRT, subretinal fluid, intraretinal fluid, and intraretinal cysts. In cases involving disagreement between the classifications of the two graders, they discussed the results with a third grader (ZZ) until they reached a consensus.

### Analysis of CNV area, vessel area, GVC, and GLD with OCTA

**Fig. 1:**
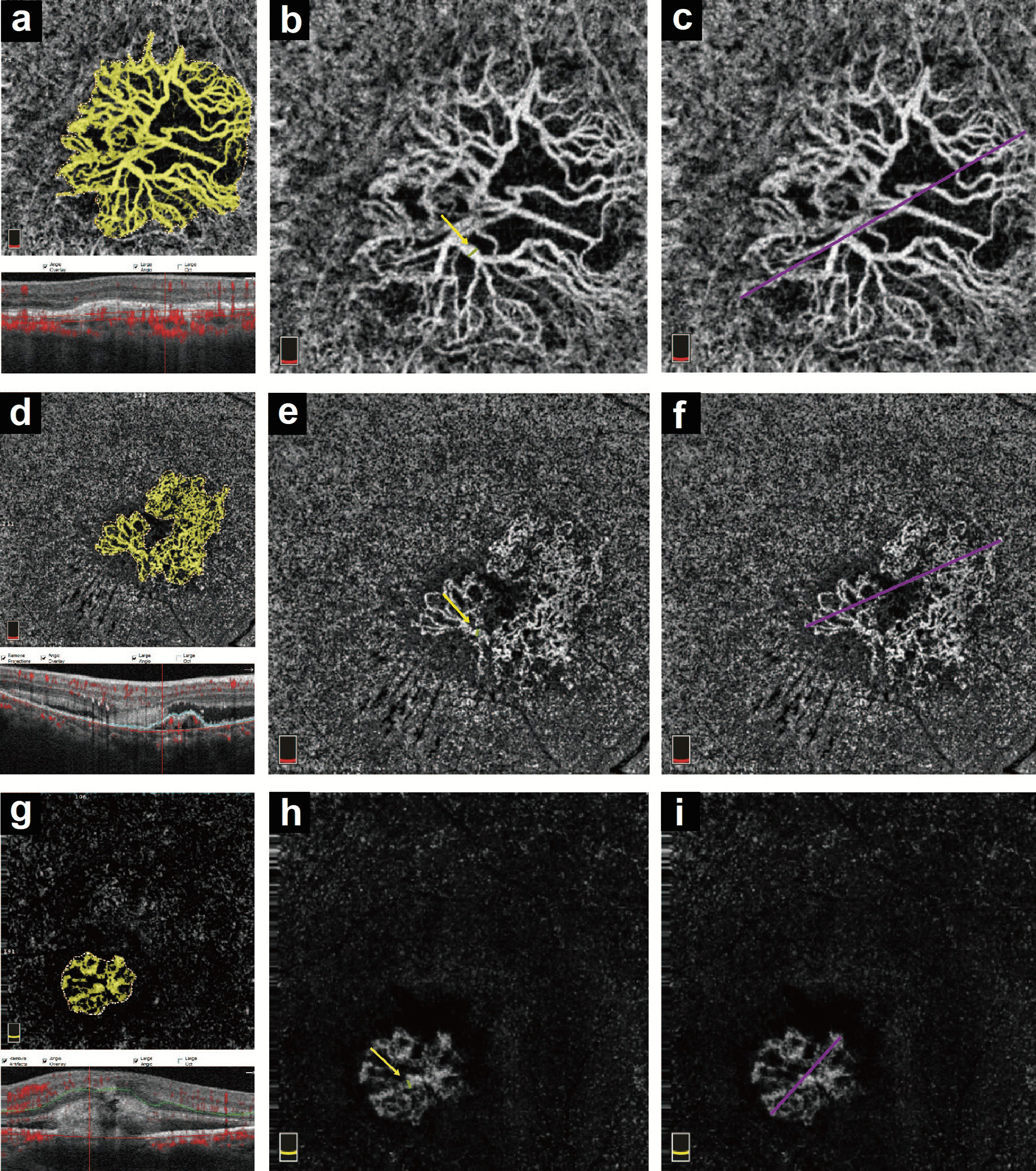
The OCTA parameters measured in all types of CNV. A 3 × 3 mm optical coherence tomography angiography (OCTA) automatic segmented choriocapillaris layer showed images of a 74-year-old male type 1 patient (**a**–**c**); A 67-year-old male mixed type patient (**d**–**f**); A 78-year-old female type 2 patient (**g**–**i**). Lesion’s size on OCTA choriocapillaris layer (shown in yellow) and the neovascular complex size was computed by AngioAnalytic System. Greatest vascular caliber (GVC) and greatest linear dimension (GLD) were manually measured on the OCTA choriocapillaris layer

The measurement of CNV lesion size was performed as described by Kuehlewein et al. [20] using the newest AngioAnalytics System (software version 2017.1.0.151). Briefly, the “flow area” function allowed the quantification of a user-defined vascular area, followed by automatic computation of “select area” and “vessel area”. The “select area” represented the total CNV area, while the “vessel area” represented the total area of solely detectable vessels determined by AngioVue software within the user-defined region. To correct for automated segmentation errors and projection artifacts of OCTA, the segmentation of the outer retina and choriocapillaris was manually adjusted by two masked graders (BL and FY) to show the optimal visualization of the CNV lesion for quantitative assessment (Fig. 1a, d, g). The measurement of the GVC was performed as described by Spaide RF. [21]. Briefly, GVC was defined as the caliber of the main trunk or the largest vessel enclosed by the lesion area after manual adjustment of the segmentation to produce a distinct feeder trunk using OCTA, and it was measured using ImageJ (National Institutes of Health, Bethesda, Maryland, USA) (Fig. 1b, e, h). The measurement of the GLD was described by Muakkassa et al. [22]. Briefly, the GLD was defined as the greatest distance enclosed by the CNV lesion area. After the lesion area was manually outlined by the readers using OCTA, GLD was measured using ImageJ (National Institutes of Health, Bethesda, MD, USA) (Fig. 1c, f, i).

Intergrader agreement between the two masked graders was assessed using intraclass correlation (ICC) values for "select area", "vessel area", GVC, and GLD. Any difference greater than 30% between graders was resolved by open discussion. If no consensus could be reached, they discussed the results with a senior reviewer (XDS) until the discrepancy was resolved. The yellow-colored region is shown in Fig. 1.

### Statistical analyses

Categorical data were presented as frequency counts and percentages, and the values of all continuous variables were presented as mean ± standard deviation. Comparisons of patient characteristics between responders and non-responders were performed using Pearson’s Chi-squared or Fisher’s exact test (discontinuous variable), Student’s t-test, or Mann–Whitney U test (continuous variables), as appropriate. Associations between the response to anti-VEGF agents and possible features were evaluated using logistic regression analysis. The sample size was mainly determined based on the number of covariates in the logistic model [26, 27]. Thus, we included 60 patients who met the criteria in our study. Statistical analyses were performed using the SPSS software (version 20.0; IBM, Armonk, NY, USA). Statistical significance was set at *P* < 0.05.

## Results

### Baseline characteristics

The baseline characteristics of the 60 eyes with active nAMD in the 60 patients are summarized in Table 1. The mean age of the participants was 70.8 ± 9.6 years. Mean BCVA was 50.8 ± 21.4 ETDRS letters at baseline. The median and interquartile range of the duration of symptoms was 5.0 (1.0, 12.0) weeks. Each eye was treated with a loading phase of three consecutive monthly injections, and then underwent PRN dosing at the Eye Center of Shanghai General Hospital.

**Table 1:** Demographics and clinical data of age-related macular degeneration patients

Parameters	Values	Responders	Non-responders	*P* value
N	60	39	21	
Patient age (years)	70.8 ± 9.6	71.8 ± 8.9	68.9 ± 10.7	0.256
Sex, N (%)				0.416
Male	35 (58.3)	21 (53.8)	14 (66.7)	
Female	25 (41.7)	18 (46.2)	7 (33.3)	
Type of CNV				0.009
Type 1, N (%)	32 (53.3)	16 (41.0)	16 (76.2)	
Type 2, N (%)	11 (18.3)	11 (28.2)	0 (0.0)	
Mixed type 1 and 2, N (%)	17 (28.3)	12(30.8)	5 (23.8)	
Baseline BCVA (ETDRS letters) (mean ± SD)	50.8 ± 21.4	50.0 ± 21.4	52.3 ± 21.8	0.691
Duration of symptoms (weeks) (median, IQR)	5.0 (1.0, 12.0)	5.0 (1.0, 12.0)	7.0 (2.0, 12.0)	0.462
Mean number of injections at 6 months	4.12 ± 1.03	4.03 ± 1.05	4.28 ± 1.02	0.931

*CNV* = choroidal neovascularization; *BCVA* = best-corrected visual acuity; *SD* = standard deviation; *IQR* = interquartile range

### Reproducibility of OCTA characteristics

Two blinded investigators independently measured the CNV area, vessel area, GVC, and GLD through OCTA. Comparison of results obtained by the two investigators showed high reproducibility, with ICC values of ≥ 0.97 for all four parameters at baseline and 3 months.

### CNV type and OCTA morphologic characteristics between responders and non-responders

Six months after the anti-VEGF therapies, among the 60 patients, 39 and 21 patients were categorized as responders and non-responders, respectively. The two groups showed no significant differences in age, sex, baseline BCVA, or duration of symptoms. Response to anti-VEGF therapy differed significantly among patients with different types of CNV (Table 1, *P* = 0.009). Specifically, among the 39 responders, 11 (28.2%) showed type 2 NV and 12 (30.8%) had mixed-type NV, showing a higher rate compared to the corresponding values in non-responders. The mean number of anti-VEGF injections 6 months after baseline showed no significant difference between responders and non-responders (Table 1).

At the baseline evaluation, the mean CNV area and vessel area in the responders and non-responders were 2.12 ± 2.67 *vs.* 2.10 ± 2.76 mm^2^ and 1.09 ± 1.30 *vs.* 1.12 ± 1.54 mm^2^, respectively. The mean GVC and GLD in the responders and non-responders were 59.2 ± 18.6 *vs.* 48.0 ± 18.8 μm and 1745.7 ± 771.8 *vs.* 1803.4 ± 1021.3 μm, respectively. Responders and non-responders showed no significant difference in baseline CNV area, vessel area, or GLD (*P* > 0.05). However, baseline GVC showed a slight difference between responders and non-responders (*P* = 0.046, Table 2).

**Table 2:** Comparison of OCTA characteristics in each group

Parameters	Responders (mean ± SD)	Non-responders (mean ± SD)	*P* value
Baseline CNV area (mm^2^)	2.12 ± 2.67	2.10 ± 2.76	0.987
Baseline vessel area (mm^2^)	1.09 ± 1.30	1.12 ± 1.54	0.947
Baseline GVC (μm)	59.2 ± 18.6	48.0 ± 18.8	0.046
Baseline GLD (μm)	1745.7 ± 771.8	1803.4 ± 1021.3	0.821
Change in CNV area (mm^2^)	− 0.28 ± 0.75	0.38 ± 1.50	0.040
Change in vessel area (mm^2^)	− 0.08 ± 0.41	0.15 ± 0.89	0.225
Change in GVC (μm)	− 4.98 ± 17.17	11.01 ± 14.10	0.001
Change in GLD (μm)	− 139.8 ± 318.7	108.5 ± 404.0	0.019

*OCTA* = optical coherence tomography angiography; *GVC* = greatest vascular caliber; *GLD* = greatest linear dimension; *SD* = standard deviation; “Changes” refers to “end of loading phase” *vs.* “baseline”

**Fig. 2:**
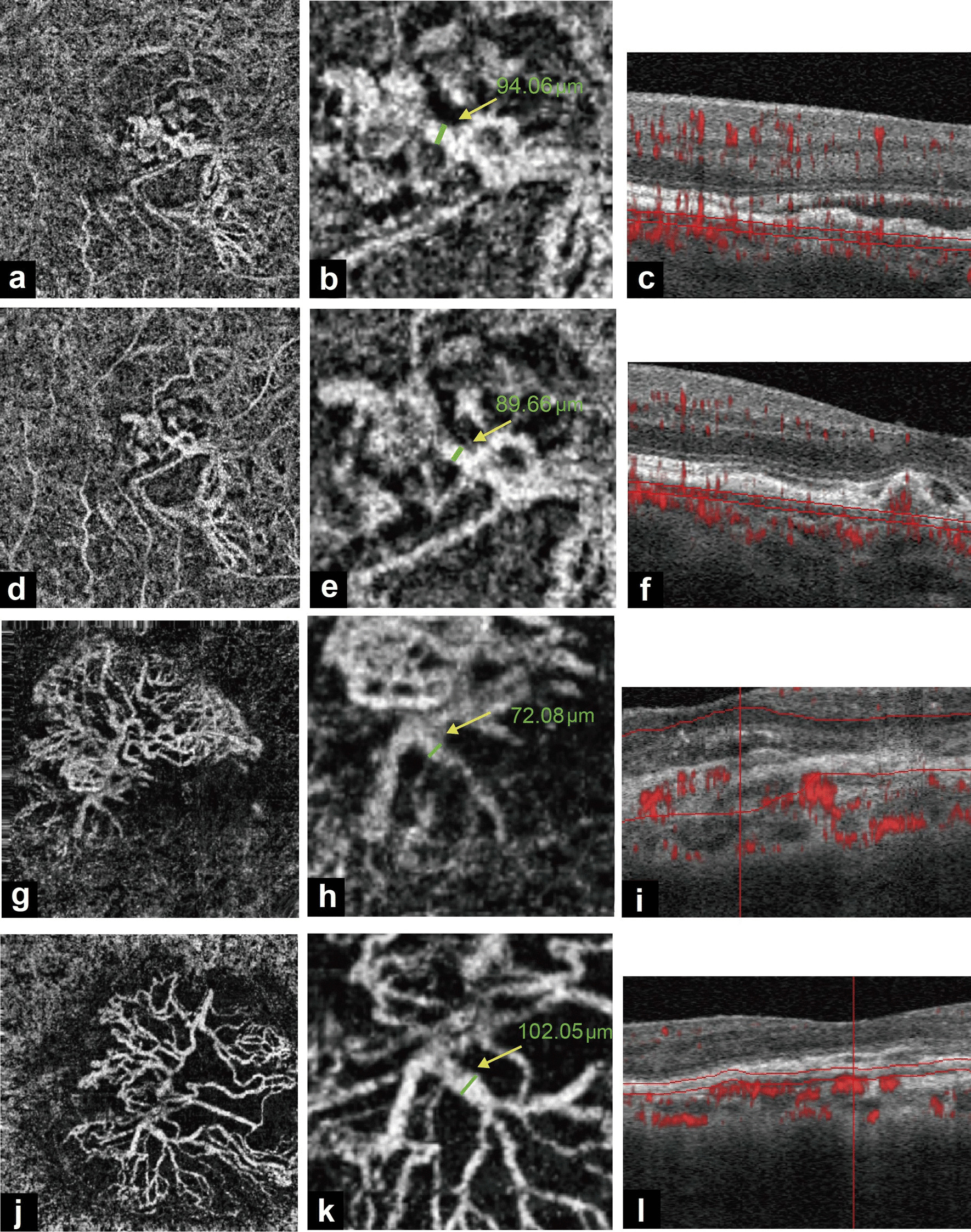
Different response to different types of anti-VEGF patients. **a**–**f** A 76-year-old male type 2 patient showing good response. Greatest vascular caliber (GVC) was 94.06 μm at baseline and decreased to 89.66 μm after three monthly anti-VEGF treatment; **g**–**l** A 75-year-old male type 1 patient demonstrating poor response. GVC was 72.08 μm at baseline and increased to 102.05 μm after three monthly anti-VEGF treatment

After the 3-month loading doses of anti-VEGF injections, responders and non-responders showed significant differences in the changes in CNV area. The CNV area in the responder group had reduced by 0.28 ± 0.75 mm^2^ in comparison with the baseline value, while the corresponding value in the non-responder group had increased by 0.38 ± 1.50 mm^2^ (*P* = 0.040). The mean vessel area of responders had decreased by 0.08 ± 0.41 mm^2^ while that of non-responders had increased by 0.15 ± 0.89 mm^2^, with no significant difference between the two groups (*P* = 0.225). The change in GVC differed significantly between responders and non-responders after loading doses of anti-VEGF agents. The GVC in responders had decreased by 4.98 ± 17.17 μm, while that in non-responders had increased by 11.01 ± 14.10 μm (*P* = 0.001, Table 2, Fig. 2). Changes in GLD showed a similar trend as the changes in GVC (*P* = 0.019, Table 2).

### Factors associated with response to anti-VEGF therapies

Factors significantly associated with the response to anti-VEGF therapies and the mean number of injections of anti-VEGF agents were included in a multivariate logistic regression model. Thus, multivariate logistic regression analysis was performed to screen for independent predictors of the response to anti-VEGF agents. Patients who showed an increase in GVC after loading doses of anti-VEGF injections were less likely to achieve a good response after 6 months (*P* = 0.008, adjusted odds ratio = 1.083; 95% CI: 1.021–1.148, Table 3).

**Table 3:** Variables influencing response to anti-VEGF therapies on logistic regression analysis

Factors	Adjusted OR	95% CI for adjusted OR	*P* value
CNV type	1.537	0.436–5.413	0.504
Number of injections	2.039	0.958–4.340	0.065
CNV area change	1.823	0.406–8.187	0.433
GVC change	1.083	1.021–1.148	0.008
GLD change	1.000	0.997–1.004	0.806
GVC baseline	1.021	0.981–1.062	0.310

*VEGF* = vascular endothelial growth factor; *OR* = odds ratio; *CI* = confidence Interval; *CNV* = choroidal neovascularization; *GVC* = greatest vascular caliber; *GLD* = greatest linear dimension; “Changes” refers to “end of loading phase” *vs.* “baseline”

## Discussion

Detection of predictors of anti-VEGF response among patients with nAMD is challenging due to the diverse clinical phenotypes and intricate pathological mechanisms underlying this disease. Previous studies have identified that many factors, including patient age, lesion characteristics, lesion duration, EZ, and subretinal hyperreflective material, are relevant to the response to anti-VEGF therapy [3, 9, 28–30]. In our study, we included data regarding CNV subtype, changes in the CNV area, GVC, and GLD observed from OCTA after 3 months of anti-VEGF injections as potential factors for predicting the 6-month prognosis of nAMD. This is because 3 initial anti-VEGF injections were proven to be the best predictive factors for favorable, long-term, and good VA by Chae et al. [29].

In our study, we found that type 2 and mixed-type NV seemed to yield a better anti-VEGF response than type 1 NV after 6 months of anti-VEGF treatment. Twenty-three cases (59%) involving responders showed type 2 or mixed-type NV, while 16 cases (76%) involving non-responders showed type 1 NV. Mettu et al. [5] comprehensively reviewed the relationship between neovascular subtypes and treatment response and found that the rate of persistent disease activity (poor response) was 25% among patients with type 2 NV but 41% among patients with type 1 NV after 1 year of treatment, which was consistent with our results. However, this might not be the same as the response to long-term treatment, especially for type 2 lesions. At the end of a 5-year treatment, Daniel et al. [31, 32] found that patients with type 2 NVs (e.g., predominantly classic CNV) were at a higher risk of developing scars and poor visual acuity outcomes than patients with other subtypes, regardless of the drug or treatment regimen. Furthermore, OCT showed that type 2 NV disrupted the RPE layer and was more likely to cause loss of photoreceptor outer segments, ELM, and EZ. Moreover, Mettu et al. [5] identified two new morphologic phenotypes of the pathologic new vessel in AMD, which were named as arteriolar and capillary patterns, using high-speed ICGA. In a retrospective cohort study and a prospective study, they observed that eyes with the capillary subtype were highly responsive to anti-VEGF therapy, while eyes with an arteriolar pattern exhibited persistent activity lesions in approximately 60% of cases. In addition, they observed that arteriolar pattern lesions with a high flow were more likely to exhibit poor response, suggesting that the hemodynamics of high blood flow may mediate aspects of poor response, especially persistent fluid, and hemorrhage. Together, OCTA has been shown to delineate neovascular morphology in an analogous fashion and offers a noninvasive alternative to ICGA to explore this hypothesis [20].

OCTA has been utilized to describe the characteristics of patients with type 1 and type 2 NV because it is noninvasive and allows in-depth visualization of vascular anomalies in the macula [33]. El Ameen et al. [34] reported different patterns of type 2 NV, which were named “medusa-shaped” and “glomerulus-shaped” lesions. Laura et al. [20] reported a similar morphological pattern of CNV in type 1 NV, and Eliana et al. [16] analyzed type 1 NV lesion size by using OCTA. However, few studies have analyzed the differences before and after anti-VEGF therapy among different types of CNV by OCTA. These facts led us to further analyze the characteristics of OCTA in different types of CNV and investigate their correlation with the response to anti-VEGF therapy. In the present study, we compared diverse OCTA parameters of treatment-naïve CNV patients in patients showing different anti-VEGF responses. However, none of the baseline OCTA parameters, except for GVC, showed significant differences between responders and non-responders: the baseline GVC in responders was slightly thicker than that in non-responders. This result was similar to those described in other studies on patients with type 1 NV [16, 35]. Laura et al. previously reported that the GVC in type 1 NV was larger than that observed in our study. This might be because the patients in their study had received an average of 15.3 intravitreal anti-VEGF injections before enrollment. Consistent with this assumption, the study by Spaide et al. demonstrated that repeated intravitreal anti-VEGF injections might accelerate the maturation of CNV trunk vessels, which made the GVC of the vessels larger than that of the treatment-naïve patients in our study [21].

More importantly, our findings revealed that a reduction in GVC at 3 months after treatment with anti-VEGF agents was an independent prognostic factor for a good response after 6 months. The potential reasons for this finding have been discussed by Bellou et al. [36], who suggested that in CNV, the major central trunk of the neovascular complexes may be more resistant to anti-VEGF treatment because of the protective pericytes blanketing the endothelial cells. This finding can also partly explain why type 2 and mixed-type NV seemed to show better anti-VEGF responses. Kuehlewein et al. [20] observed a greater likelihood of large mature neovascular complexes in type 1 NV eyes than in type 2 NV eyes, indicating a greater likelihood of poor anti-VEGF responses. Therefore, identification of the diversity of the maturation processes of the main trunk of neovascular complexes through OCTA can facilitate a better assessment of the role of anti-VEGF responses in different types of CNV. In other studies, since OCTA could better estimate the real lesion size of CNV than ICG and FFA, Coscas et al. showed that the presence of tiny choroidal branching vessels and peripheral anastomotic arcades appeared to predict lesion activity [37]. Mastropasqua et al. [38] observed that smaller-caliber vessels changed 48 h after treatment, while larger trunks remained well-perfused. The vascular flow remodeling induced by recurrent anti-VEGF treatment was recently described by Miere et al., who showed that anti-VEGF treatment seemed to be effective against newly formed capillaries, with the “pruned vascular tree” pattern characterized by a thick matured main vascular trunk with no thin ramifications appearing to be prominent after several anti-VEGF agents [39]. Therefore, we hypothesized that the changes in the main trunk vessel of CNV after the loading doses of anti-VEGF agents, and not any other neovascular area or tiny branching vessels visualized on OCTA, could predict the long-term response to anti-VEGF therapy. These results together suggest that GVC variations under OCTA, along with CNV type, could be potential biomarkers for estimating long-term anti-VEGF response and facilitating the selection of an optimal regimen after the loading doses of anti-VEGF injections. Focusing on GVC variations after a loading dose of anti-VEGF injections may supplement the current retreatment criteria and help optimize the treatment regimen for nAMD.

In addition, the disruption or recovery of the EZ in SD-OCT has been confirmed to be associated with the prognosis of nAMD patients [40–42], and in our study, the GVC, which was a parameter observed in OCTA, has been proven to be associated with the prognosis of nAMD patients. Therefore, the potential relationship between the results obtained using OCTA and those obtained with SD-OCT is worth studying. Liu et al. found that patients with retinitis pigmentosa and high choroidal vessel density measured using noninvasive OCTA were likely to have longer EZs, better vision, and lower visual acuity. [43] Hence, we assumed that CNV shrinkage or reduction in GVC might be beneficial to the recovery of EZ to some extent.

Our study has a few limitations. First, due to the low incidence of type 3 NV (RAP), our study only included patients with type 1, type 2, and mixed-type NV. The features of type 3 NV on OCTA and their correlation with the response to anti-VEGF therapy require further study. Second, since segmentation in OCTA may not cover all CNV lesions, some parts of the CNV may be missed. Further refinement of this novel technique will improve its clinical applicability. Third, our study focused on the initial morphologic changes after loading doses based on OCTA to predict the 6-month anti-VEGF response. The ability of these changes to predict long-term anti-VEGF responses needs further investigation.

## Conclusions

In summary, type 2 and mixed-type NV showed better responses to anti-VEGF therapies than type 1 NV. Changes in GVC after 3 months of treatment were significantly associated with the response to anti-VEGF therapies at 6 months, suggesting that GVC shrinkage might be a biomarker for predicting the response to anti-VEGF therapies. Newer anti-VEGF drugs designed with smaller molecular weights and the ability to penetrate the RPE to maintain a high drug concentration gradient in the retina will be favorable for the treatment of nAMD.

## Data Availability

All data generated or analyzed during this study are included in this article. Further enquiries can be directed to the corresponding author.
